# 

**Published:** 1840-01

**Authors:** 


### BRITISH AND FOREIGN

### MEDICAL REVIEW

OR

### QUARTERLY JOURNAL

OF

### PRACTICAL MEDICINE AND SURGERY

---

EDITED BY

JOHN FORBES M.D. F.R.S.

---

S.G.O. INDEXED

VOL. IX.

JANUARY—APRIL

Surgeon Genl's Office LIBRARY. 34532 Washington, D.C.

LONDON
JOHN CHURCHILL PRINCES STREET SOHO
MDCCCXL.

W
1
BR34

LONDON:

PRINTED BY C. ADLARD, BARTHOLOMEW CLOSE.

### BOOKS RECEIVED FOR REVIEW.

ENGLISH.

1. First Annual Report of the Registrar-General of Births, Deaths, and Marriages. Presented to both Houses of Parliament by command of her Majesty.—London, 1839. Folio, pp. 16. Abstracts and Appendix, pp. 77.

2. Observations on Malaria, with suggestions for ascertaining its nature. By Thomas Hopkins.—Manchester, 1839. 8vo, pp. 26.

3. Changes produced in the Nervous System by Civilization, &c. By R. Verity, M.D. Second Edition.—Paris, 1839. 8vo, pp. 143. 6s.

4. The Anatomist's Manual; or, a Treatise on the manner of preparing all the parts of Anatomy, followed by a complete description of these parts. By J. P. Maygrier, M.D.P. Translated from the latest French Edition.—London, 1839. 8vo, pp. 564.

5. Text-book of Anatomy for Junior Students. By Alex. Jardine Lizars, M.D., &c.—Edinb. (no date). 12mo, pp. 272. 6s.

6. Cases of Chronic Hydrocephalus, or Water in the Head; with observations, and a detail of a new and successful plan of Cure. By J. F. Barnard, M.R.C.S.—Bath, 1839. 8vo, pp. 30.

7. Clinical Remarks on some cases of Liver Abscess presenting externally. By J. G. Malcolmson, M.D. (from the Edinb. Journ. No. CXLI.)—Edin. 1839. 8vo, pp. 44.

8. Medical Portrait Gallery. Part XX. XXI. XXII. Containing William Harvey, M.D.; Marshall Hall, M.D.; William Cullen, M.D.; Sir James M'Grigor, Bart.; John Brown, M.D.; Richard Powell, M.D.

9. Practical Observations, showing that Mercury is the sole cause of what are termed secondary symptoms. By P. J. Murphy, M.D.—Lond. 1839. 8vo, pp. 106.

10. The Modern Treatment of Syphilitic Diseases, both primary and secondary; comprising an account of the new remedies, with numerous formulæ. By Langston Parker, Lecturer on Anatomy, &c., in the Birmingham School.—London, 1839.—Sm. 8vo, pp. 158. 5s.

11. Observations on Malaria, with suggestions for ascertaining its nature. By Thomas Hopkins.—Manchester, 1839. 8vo, pp. 26.

12. Insecta. By George Newport, Esq. (From the Cyclopædia of Anatomy and Physiology.)—London, 1839. 8vo, pp. 128.

13. Elements of Physiology. By J. Müller, M.D., &c. Translated by W. Baly, M.D. Second Edition. Part I.—London, 1839. 8vo, pp. 471. 10s.

14. Valedictory Address to the Students of Medicine of the University of New York. Delivered Feb. 28, 1839. By J. B. Beck, M.D.—New York, 1839. 8vo, pp. 24.

15. A Practical Treatise on the Diseases of the Eye. By William Mackenzie, M.D., Surgeon-Oculist to Her Majesty, &c.—London, 8vo, pp. 923. 25s.

16. A Treatise on the Medical Jurisprudence of Insanity. By I. Ray; with an Introductory Essay by Dr. Spillan, M.D.—London, 1839. 8vo, pp. 436.

17. Treatise on Obstetric Auscultation. By Dr. H. F. Naegele. Translated from the German, by Charles West, M.D.—London, 1839. 12mo, pp. 120.

18. Observations on Yaws, and its influence in originating Leprosy; also Observations on Acute Traumatic Tetanus and Tetanus Infantum. By James Maxwell, M.D.—Edin. 1839. 8vo, pp. 134.

19. Illustrations of Osteology. By T. S. G. Boisragon, M.D. Folio. Two Plates. 1839,

20. A Treatise on the Diseases of Infants. By C. M. Billard, M.D., with Notes by Dr. Ollivier, of Angers. Translated from the Third French Edition, with an Appendix. By James Stewart, M.D.—London, 1839. 8vo, pp. 620.

21. Elements of Natural Philosophy; being an experimental introduction to the study of the Physical Sciences. By Golding Bird, M.D., F.L.S., F.G.S., &c. &c.—London, 1839. 8vo, pp. 407.

22. Gatherings from Grave-Yards, particularly those of London; with a concise history of the modes of interment among different nations from the earliest periods, &c. &c. By G. A. Walker, Surgeon.—London, 1839. 8vo, pp. 258.

23. The Fifty-first Report of the Visiting Justices of the County Lunatic Asylum at Hanwell, with the Report of the Resident Physician, John Conolly, M.D.—London, 1839. 8vo, pp. 70.

24. The Eye: A Treatise on the art of preserving this organ in a healthy condition, and of improving the sight, &c. By J. C. A. Franz, Doctor of Medicine, Surgeon of the University of Leipzic, &c. —London, 1839. 8vo, pp. 296.

25. Medical Report of the House of Recovery and Fever Hospital, Cork Street, Dublin, for two years, from 1st January, 1837, to 31st December, 1838. By G. A. Kennedy, M.D., Physician to the House of Recovery, &c.—Dublin, 1839. 8vo, pp. 120.

26. Elements of Surgery. By Robert Liston, Surgeon to the North London Hospital, &c. Second Edition. Illustrated with Engravings.—London, 1840. 8vo, pp. 795.

27. The Retrospective Address delivered at the Seventh Anniversary Meeting of the Provincial Medical and Surgical Association, held at Liverpool, July 24th-5th, 1839. By J. A. Symonds, M.D., Senior Physician to the Bristol Hospital.—Worcester, 1839. 8vo, pp. 100.

28. An Introductory Lecture on the Anatomy, Physiology, and Diseases of the Eye, delivered at the Birmingham School of Medicine, October 4, 1839. By Richard Middlemore, Surgeon to the Eye Infirmary.—London, 1839. 8vo, pp.

29. Observations on Medical Education, with a view to legislative interference. By Richard Jones, M.R.C.S.—London, 1839. 8vo, pp. 52.

30. A Lecture introductory to the Course on the Principles and Practice of Medicine, delivered at the Aldersgate School, Oct. 1839. By Robert Willis, M.D., &c.—London, 1839. 8vo, pp. 21.

FOREIGN.

1. Beiträge zur operativen Orthopädie. oder Erfahrungen über die subcutane Durchschneidung verkürzen Muskeln und derer Sehnen. Von Dr. L. Stromeyer. Mit 8 lith. Tafeln.—Hannover, 1838. 8vo, pp. 154.

2. Beobachtungen auf dem Gebiete der Pathologie und Pathologischen Anatomie. Von Dr. J. F. H. Albers.—Bonn, 1838. 8vo, pp. 218.

3. Beobachtungen und Erfahrungen aus dem Gebiete der praktischen Arznei- und Wundarzneikunst. Von Dr. Löwenhardt.—Prenzlau, 1838. 8vo, pp. 425.

4. Memorie della Società Medico-chirurgica di Bologna. Vol. ii. Fasc. ii —Bologna, 1839. 8vo.

5. Das Blut in seiner heilthäthigen Beziehung zum Schmerz im Allgemeinen und zu den (wahren und unwahren) Neuralgien inbesondere. Zur vorläufigen Erörterung empfohlen von C. J. Heidler, M.D. &c.—Prag. 1839. 8vo, pp. 57.

6. Osservazioni sul sangue umano, e Considerazioni sui metodi di piu' conveniente investigazione intorno ai fenomeni dei corpi organici Lettera Di Maurizio Bufalini.—Venezia, 1838. 8vo, pp. 123.

7. Six Mois de Séjour en Angleterre pendant l'année 1836. Par Sirus Pirondi, M.D.—Marseille, 1839. 8vo, pp. 435.

8. Avvisi agli Stranieri che amano di viaggiare in Italia o dimorarvi per conservare o recuperare la salute. Del Professore Giacomo Barzellotti, dell' I. e R. Università di Pisa.—Firenze, 1838. 8vo, pp. 290.

9. Théorie de la Phlogose, de J. Rasori. Traduite de l'Italien, par Sirus Pirondi, M.D.—Paris, 1839. 2 Tom. 8vo, pp. 334-406.

10. De Strabismo Dissertatio, quam scripsit N. G. Melchior, M.D.—Havniæ, 1839. 8vo, pp. 75.

11. De Crimine Raptûs, dissertatio quam scripsit F. C. Bornemann, Juris Candidatus.—Havniæ, 1839. 8vo, pp. 136.

12. Conspectus Aegrotorum qui in 1837-8, in Nosocomio Kiliensi tractati sunt. Auctore O. H. With, M.D.—Kiliæ, 1839. 4to, pp. 38.

13. Commentationis de Arsenico Specimen. Ad summos in philosophiæ honores obtinendos, scripsit B. S. Lewy.—Berolini, 1839. 8vo, pp. 45.

14. Dissertatio de Radesyge, Lepra et Elephantiasi Septentrionali. Auctore J. J. Hialtalino.—Kiliæ, 1839. 8vo, pp. 36.

15. De Chemicis Calculorum Vesicariorium rationibus. Scripsit E. A. Scharling, Chemiæ Lector.—Havniæ, 1839. 4to, pp. 52. Cum Tab. vi.

16. Londres Ancien et Moderne, ou Recherches sur l'état physique et social de cette metropole. Par A. M. Bureaud-Rioffrey, M.D., &c.—Paris et Londres, 1839. 8vo, pp. 138

17. Histoire de la Lithotritie précedée de reflexions sur la dissolution des calculs urinaires. Par Leroy-D'Etiolles, M.D.—Paris, 1839, 8vo, pp. 168.

18. Dissertazioni Hahnemanniane con Annotazioni critiche.—Cremona, 1839. 8vo, pp. 458.

19. Tarantismo o Malattia prodotta dalle Tarantole velenose. Memoria di Achille Vergari, M.D.—Napoli, 1839. 8vo, pp. 61.

20. Etudes statistiques sur les Fractures et les Luxations. Par M. Malgaigne, Professeur de la Faculté, &c.—Paris, 1839. 8vo, pp. 31.

